# Supplementary material for: Integration of metataxonomic data sets into microbial association networks highlights shared bacterial community dynamics in fermented vegetables
Source: Microbiol Spectr. 2024 May 15;12(6):e00312-24. doi: 10.1128/spectrum.00312-24 (PMC11237590; doi:10.1128/spectrum.00312-24)
Supplement: Supplemental material — Table S1; Fig. S1-S4. [file spectrum.00312-24-s0001.pdf]

Integration of metataxonomic datasets into microbial association networks highlights shared bacterial community dynamics in fermented vegetables

Romane Junker, Florence Valence, Michel-Yves Mistou,
Stéphane Chaillou, Hélène Chiapello

List of supplemental materials

- Table S1: page 2
- Figure S1: page 5
- Figure S2: page 6
- Figure S3: page 7
- Figure S4: page 8

Table S1: Description of the ASVs belonging to SBM cluster 5

Name	Study prevalence	Order	Genus	Sequence
ASV557	9	Lactobacillales	Leuconostoc	TACGTATGTCCCGAGCGTTATCCGGATTTATTGGGCGTAAAGCGAGCGCAGACGGTTGATTAAGTCTGATGTGAAAGCCCGGAGCTCAACTCCGGAATGGCATTGGAAACTGGTTAACTTGAGTGTTGTAGAGGTAAGTGGAACTCCATGTGTAGCGGTGGAATGCGTAGATATATGGAAGAACACCAGTGGCGAAGGCGGCTTACTGGACAACAACTGACGTTGAGGCTCGAAAGTGTGGGTAGCAAACAGG
ASV23	9	Lactobacillales	Weissella	TACGTATGTTCCAAGCGTTATCCGGATTTATTGGGCGTAAAGCGAGCGCAGACGGTTATTTAAGTCTGAAGTGAAAGCCCTCAGCTCAACTGAGGAATTGCTTTGGAAACTGGATGACTTGAGTGCAGTAGAGGAAAGTGGAACTCCATGTGTAGCGGTGAAATGCGTAGATATATGGAAGAACACCAGTGGCGAAGGCGGCTTTCTGGACTGTAACTGACGTTGAGGCTCGAAAGTGTGGGTAGCAAACAGG
ASV22	9	Lactobacillales	Latilactobacillus	TACGTAGGTGGCAAGCGTTGTCCGGATTTATTGGGCGTAAAGCGAGCGCAGGCGGTTTCTTAAGTCTGATGTGAAAGCCTTCGGCTCAACCGAAGAAGTGCATCGGAAACTGGGAAACTTGAGTGCAGAAGAGGACAGTGGAACTCCATGTGTAGCGGTGAAATGCGTAGATATATGGAAGAACACCAGTGGCGAAGGCGGCTGTCTGGTCTGTAACTGACGCTGAGGCTCGAAAGCATGGGTAGCAAACAGG
ASV1	9	Lactobacillales	Lactiplantibacillus	TACGTAGGTGGCAAGCGTTGTCCGGATTTATTGGGCGTAAAGCGAGCGCAGGCGGTTTTTTAAGTCTGATGTGAAAGCCTTCGGCTCAACCGAAGAAGTGCATCGGAAACTGGGAAACTTGAGTGCAGAAGAGGACAGTGGAACTCCATGTGTAGCGGTGAAATGCGTAGATATATGGAAGAACACCAGTGGCGAAGGCGGCTGTCTGGTCTGTAACTGACGCTGAGGCTCGAAAGTATGGGTAGCAAACAGG
ASV9	9	Lactobacillales	Leuconostoc	TACGTATGTCCCGAGCGTTATCCGGATTTATTGGGCGTAAAGCGAGCGCAGACGGTTTATTAAGTCTGATGTGAAAGCCCGGAGCTCAACTCCGGAATGGCATTGGAAACTGGTTAACTTGAGTGCAGTAGAGGTAAGTGGAACTCCATGTGTAGCGGTGGAATGCGTAGATATATGGAAGAACACCAGTGGCGAAGGCGGCTTACTGGACTGCAACTGACGTTGAGGCTCGAAAGTGTGGGTAGCAAACAGG
ASV61	8	Lactobacillales	Enterococcus	TACGTAGGTGGCAAGCGTTGTCCGGATTTATTGGGCGTAAAGCGAGCGCAGGCGGTTTCTTAAGTCTGATGTGAAAGCCCCCGGCTCAACCGGGGAGGGTCATTGGAAACTGGGAGACTTGAGTGCAGAAGAGGAGAGTGGAATTCCATGTGTAGCGGTGAAATGCGTAGATATATGGAGGAACACCAGTGGCGAAGGCGGCTCTCTGGTCTGTAACTGACGCTGAGGCTCGAAAGCGTGGGGAGCGAACAGG
ASV92	8	Enterobacterales	Yersinia	TACGGAGGGTGCAAGCGTTAATCGGAATTACTGGGCGTAAAGCGCACGCAGGCGGTTTGTTAAGTCAGATGTGAAATCCCCGCGCTTAACGTGGGAACTGCATTTGAAACTGGCAAGCTAGAGTCTTGTAGAGGGGGGTAGAATTCCAGGTGTAGCGGTGAAATGCGTAGAGATCTGGAGGAATACCGGTGGCGAAGGCGGCCCCCTGGACAAAGACTGACGCTCAGGTGCGAAAGCGTGGGGAGCAAACAGG
ASV159	8	Enterobacterales	*NA*	TACGGAGGGTGCAAGCGTTAATCGGAATTACTGGGCGTAAAGCGCACGCAGGCGGTTTGTTAAGTCAGATGTGAAATCCCCGAGCTTAACTTGGGAACTGCATTTGAAACTGGCAAGCTAGAGTCTTGTAGAGGGGGGTAGAATTCCAGGTGTAGCGGTGAAATGCGTAGAGATCTGGAGGAATACCGGTGGCGAAGGCGGCCCCCTGGACAAAGACTGACGCTCAGGTGCGAAAGCGTGGGGAGCAAACAGG
ASV546	7	Lactobacillales	Leuconostoc	TACGTATGTCCCGAGCGTTATCCGGATTTATTGGGCGTAAAGCGAGCGCAGACGGTTGATTAAGTCTGATGTGAAAGCCCGGAGCTCAACTCCGGAATGGCATTGGAAACTGGTTAACTTGAGTGCAGTAGAGGTAAGTGGAACTCCATGTGTAGCGGTGGAATGCGTAGATATATGGAAGAACACCAGTGGCGAAGGCGGCTTACTGGACTGTAACTGACGTTGAGGCTCGAAAGTGTGGGTAGCAAACAGG
ASV21	7	Enterobacterales	Citrobacter	TACGGAGGGTGCAAGCGTTAATCGGAATTACTGGGCGTAAAGCGCACGCAGGCGGTCTGTCAAGTCGGATGTGAAATCCCCGGGCTCAACCTGGGAACTGCATCCGAAACTGGCAGGCTAGAGTCTTGTAGAGGGGGGTAGAATTCCAGGTGTAGCGGTGAAATGCGTAGAGATCTGGAGGAATACCGGTGGCGAAGGCGGCCCCCTGGACAAAGACTGACGCTCAGGTGCGAAAGCGTGGGGAGCAAACAGG
ASV10	7	Lactobacillales	Levilactobacillus	TACGTAGGTGGCAAGCGTTGTCCGGATTTATTGGGCGTAAAGCGAGCGCAGGCGGTTTTTTAAGTCTGATGTGAAAGCCTTCGGCTTAACCGGAGAAGTGCATCGGAAACTGGGAGACTTGAGTGCAGAAGAGGACAGTGGAACTCCATGTGTAGCGGTGGAATGCGTAGATATATGGAAGAACACCAGTGGCGAAGGCGGCTGTCTAGTCTGTAACTGACGCTGAGGCTCGAAAGCATGGGTAGCGAACAGG
ASV13	6	Staphylococcales	Staphylococcus	TACGTAGGTGGCAAGCGTTATCCGGAATTATTGGGCGTAAAGCGCGCGTAGGCGGTTTCTTAAGTCTGATGTGAAAGCCCACGGCTCAACCGTGGAGGGTCATTGGAAACTGGGAAACTTGAGTGCAGAAGAGGAAAGTGGAATTCCATGTGTAGCGGTGAAATGCGCAGAGATATGGAGGAACACCAGTGGCGAAGGCGACTTTCTGGTCTGTAACTGACGCTGATGTGCGAAAGCGTGGGGATCAAACAGG
ASV573	6	Pseudomonadales	Pseudomonas	TACAGAGGGTGCAAGCGTTAATCGGAATTACTGGGCGTAAAGCGCGCGTAGGTGGTTTGTTAAGTTGAATGTGAAATCCCCGGGCTCAACCTGGGAACTGCATCCAAAACTGGCAAGCTAGAGTATGGTAGAGGGTAGTGGAATTTCCTGTGTAGCGGTGAAATGCGTAGATATAGGAAGGAACACCAGTGGCGAAGGCGACTACCTGGACTGATACTGACACTGAGGTGCGAAAGCGTGGGGAGCAAACAGG
ASV133	6	Enterobacterales	*NA*	TACGGAGGGTGCAAGCGTTAATCGGAATTACTGGGCGTAAAGCGCACGCAGGCGGTCTGTCAAGTCGGATGTGAAATCCCCGGGCTCAACCTGGGAACTGCATTCGAAACTGGCAGGCTGGAGTCTTGTAGAGGGGGGTAGAATTCCAGGTGTAGCGGTGAAATGCGTAGAGATCTGGAGGAATACCGGTGGCGAAGGCGGCCCCCTGGACAAAGACTGACGCTCAGGTGCGAAAGCGTGGGGAGCAAACAGG
ASV547	6	Lactobacillales	Weissella	TACGTATGTCCCAAGCGTTATCCGGATTTATTGGGCGTAAAGCGAGCGCAGACGGTTATTTAAGTCTGAAGTGAAAGCCCTCAGCTCAACTGAGGAATTGCTTTGGAAACTGGATGACTTGAGTGCAGTAGAGGAAAGTGGAACTCCATGTGTAGCGGTGAAATGCGTAGATATATGGAAGAACACCAGTGGCGAAGGCGGCTTTCTGGACTGTAACTGACGTTGAGGCTCGAAAGTGTGGGTAGCAAACAGG
ASV2475	5	Lactobacillales	Lactococcus	TACGTAGGTCCCGAGCGTTGTCCGGATTTATTGGGCGTAAAGCGAGCGCAGGTGGTTTATTAAGTCTGGTGTAAAAGGCAGTGGCTCAACCATTGTATGCATTGGAAACTGGTAGACTTGAGTGCAGGAGAGGAGAGTGGAATTCCATGTGTAGCGGTGAAATGCGTAGATATATGGAGGAACACCGGTGGCGAAAGCGGCTCTCTGGCCTGTAACTGACACTGAGGCTCGAAAGCGTGGGGAGCAAACAGGA

ASV2461	5	Lactobacillales	Lactococcus	TACGTAGGTCCCAAGCGTTGTCCGGATTTATTGGGCGTAAAGCGAGCGCAGGTGGTTTCTTAAGTCTGATGTAAAAGGCAGTGGCTCAACCATTGTGTGCATTGGAAACTGGGAGACTTGAGTGCAGGAGAGGAGAGTGGAATTCCATGTGTAGCGGTGAAATGCGTAGATATATGGAGGAACACCGGAGGCGAAAGCGGCTCTCTGGCCTGTAACTGACACTGAGGCTCGAAAGCGTGGGGAGCAAACAGGA
ASV168	5	Lactobacillales	Vagococcus	TACGTAGGTGGCAAGCGTTGTCCGGATTTATTGGGCGTAAAGCGAGCGCAGGCGGTCTTTTAAGTCTGATGTGAAAGCCCTCGGCTCAACCGAGGAAGGTCATTGGAAACTGGAGGACTTGAGTGCAGAAGAGGAGAGTGGAATTCCATGTGTAGCGGTGAAATGCGTAGATATATGGAGGAACACCAGTGGCGAAGGCGACTCTCTGGTCTGTAACTGACGCTGAGGCTCGAAAGCGTGGGGAGCAAACAGG
ASV52	5	Enterobacterales	*NA*	TACGGAGGGTGCAAGCGTTAATCGGAATTACTGGGCGTAAAGCGCACGCAGGCGGTCTGTCAAGTCGGATGTGAAATCCCCGGGCTCAACCTGGGAACTGCATCCGAAACTGGCAGGCTTGAGTCTCGTAGAGGGGGGTAGAATTCCAGGTGTAGCGGTGAAATGCGTAGAGATCTGGAGGAATACCGGTGGCGAAGGCGGCCCCCTGGACGAAGACTGACGCTCAGGTGCGAAAGCGTGGGGAGCAAACAGG
ASV4	5	Lactobacillales	Pediococcus	TACGTAGGTGGCAAGCGTTATCCGGATTTATTGGGCGTAAAGCGAGCGCAGGCGGTCTTTTAAGTCTAATGTGAAAGCCTTCGGCTCAACCGAAGAAGTGCATTGGAAACTGGGAGACTTGAGTGCAGAAGAGGACAGTGGAACTCCATGTGTAGCGGTGAAATGCGTAGATATATGGAAGAACACCAGTGGCGAAGGCGGCTGTCTGGTCTGCAACTGACGCTGAGGCTCGAAAGCATGGGTAGCGAACAGG
ASV561	5	Enterobacterales	*NA*	TACGGAGGGTGCAAGCGTTAATCGGAATTACTGGGCGTAAAGCGCACGCAGGCGGTCTGTCAAGTCAGATGTGAAATCCCCGGGCTTAACCTGGGAACTGCATTTGAAACTGGCAGGCTTGAGTCTCGTAGAGGGGGGTAGAATTCCAGGTGTAGCGGTGAAATGCGTAGAGATCTGGAGGAATACCGGTGGCGAAGGCGGCCCCCTGGACGAAGACTGACGCTCAGGTGCGAAAGCGTGGGGAGCAAACAGG
ASV8	5	Enterobacterales	Pseudocitrobacter	TACGGAGGGTGCAAGCGTTAATCGGAATTACTGGGCGTAAAGCGCACGCAGGCGGTTTGTTAAGTCAGATGTGAAATCCCCGGGCTCAACCTGGGAACTGCATTTGAAACTGGCAAGCTTGAGTCTCGTAGAGGGGGGTAGAATTCCAGGTGTAGCGGTGAAATGCGTAGAGATCTGGAGGAATACCGGTGGCGAAGGCGGCCCCCTGGACGAAGACTGACGCTCAGGTGCGAAAGCGTGGGGAGCAAACAGG
ASV6	5	Lactobacillales	Lactococcus	TACGTAGGTCCCGAGCGTTGTCCGGATTTATTGGGCGTAAAGCGAGCGCAGGTGGTTTATTAAGTCTGGTGTAAAAGGCAGTGGCTCAACCATTGTATGCATTGGAAACTGGTAGACTTGAGTGCAGGAGAGGAGAGTGGAATTCCATGTGTAGCGGTGAAATGCGTAGATATATGGAGGAACACCGGTGGCGAAAGCGGCTCTCTGGCCTGTAACTGACACTGAGGCTCGAAAGCGTGGGGAGCAAACAGG
ASV173	5	Enterobacterales	Erwinia	TACGGAGGGTGCAAGCGTTAATCGGAATTACTGGGCGTAAAGCGCACGCAGGCGGTCTGTCAAGTCAGATGTGAAATCCCCGGGCTTAACCTGGGAACTGCATTTGAAACTGGCAGGCTAGAGTCTTGTAGAGGGGGGTAGAATTCCAGGTGTAGCGGTGAAATGCGTAGAGATCTGGAGGAATACCGGTGGCGAAGGCGGCCCCCTGGACAAAGACTGACGCTCAGGTGCGAAAGCGTGGGGAGCAAACAGG
ASV872	4	Pseudomonadales	Pseudomonas	TACGAAGGGTGCAAGCGTTAATCGGAATTACTGGGCGTAAAGCGCGCGTAGGTGGCTTGATAAGTTGGATGTGAAATCCCCGGGCTCAACCTGGGAACTGCATCCAAAACTGTCTGGCTAGAGTGCGGTAGAGGGTAGTGGAATTTCCAGTGTAGCGGTGAAATGCGTAGATATTGGAAGGAACACCAGTGGCGAAGGCGACTACCTGGACTGACACTGACACTGAGGTGCGAAAGCGTGGGGAGCAAACAGG
ASV1536	4	Lactobacillales	Vagococcus	TACGTAGGTGGCAAGCGTTGTCCGGATTTATTGGGCGTAAAGCGAGCGCAGGTGGTCTTTTAAGTCTGATGTGAAAGCCCTCGGCTCAACCGAGGAAGGTCATTGGAAACTGGAGGACTTGAGTGCAGAAGAGGAGAGTGGAATTCCATGTGTAGCGGTGAAATGCGTAGATATATGGAGGAACACCAGTGGCGAAGGCGACTCTCTGGTCTGTAACTGACACTGAGGCTCGAAAGCGTGGGGAGCAAACAGG
ASV1504	4	Lactobacillales	Weissella	TACGTATGTCCCAAGCGTTATCCGGATTTATTGGGCGTAAAGCGAGCGCAGGCGGTTATTTAAGTCTGATGTGAAAGCCTACGGCTCAACCGTAGAATTGCATCGGAAACTGGATGACTTGAGTGCAGTAGAGGAGAGTGGAACTCCATGTGTAGCGGTGAAATGCGTAGATATATGGAAGAACACCAGAGGCGAAGGCGGCTCTCTGGACTGTAACTGACGCTGAGGCTCGAAAGTGTGGGTAGCAAACAGG
ASV772	4	Enterobacterales	Serratia	TACGGAGGGTGCAAGCGTTAATCGGAATTACTGGGCGTAAAGCGCACGCAGGCGGTTTGTTAAGTCAGATGTGAAATCCCCGGGCTCAACCTGGGAACTGCATTTGAAACTGGCAAGCTAGAGTCTCGTAGAGGGGGGTAGAATTCCAGGTGTAGCGGTGAAATGCGTAGAGATCTGGAGGAATACCGGTGGCGAAGGCGGCCCCCTGGACGAAGACTGACGCTCAGGTGCGAAAGCGTGGGGAGCAAACAGG
ASV616	4	Lactobacillales	Companilactobacillus	TACGTAGGTGGCAAGCGTTGTCCGGATTTATTGGGCGTAAAGAGAATGTAGGCGGTTTATTAAGTTTGAAGTGAAAGCCCTCGGCTCAACCGAGGAAGTGCTTCGAAAACTGGTAAACTTGAGTGCAGAAGAGGAAAGTGGAACTCCATGTGTAGCGGTGGAATGCGTAGATATATGGAAGAACACCAGTGGCGAAGGCGGCTTTCTGGTCTGTAACTGACGCTGAGATTCGAAAGCATGGGTAGCAAACAGG
ASV30	4	Lactobacillales	Weissella	TACGTATGTTCCAAGCGTTATCCGGATTTATTGGGCGTAAAGCGAGCGCAGACGGTTATTTAAGTCTGAAGTGAAAGCCCTCAGCTCAACTGAGGAATGGCTTTGGAAACTGGATGACTTGAGTGCAGTAGAGGAAAGTGGAACTCCATGTGTAGCGGTGAAATGCGTAGATATATGGAAGAACACCAGTGGCGAAGGCGGCTTTCTGGACTGTAACTGACGTTGAGGCTCGAAAGTGTGGGTAGCAAACAGG
ASV570	4	Enterobacterales	*NA*	TACGGAGGGTGCAAGCGTTAATCGGAATTACTGGGCGTAAAGCGCACGCAGGCGGTTTGTTAAGTCAGATGTGAAATCCCCGGGCTCAACCTGGGAACTGCATTTGAAACTGGCAAGCTTGAGTCTTGTAGAGGGGGGTAGAATTCCAGGTGTAGCGGTGAAATGCGTAGAGATCTGGAGGAATACCGGTGGCGAAGGCGGCCCCCTGGACAAAGACTGACGCTCAGGTGCGAAAGCGTGGGGAGCAAACAGG
ASV586	3	Enterobacterales	*NA*	TACGGAGGGTGCAAGCGTTAATCGGAATTACTGGGCGTAAAGCGCACGCAGGCGGTCTGTCAAGTCGGATGTGAAATCCCCGGGCTTAACCTGGGAACTGCATTCGAAACTGGCAGGCTGGAGTCTTGTAGAGGGGGGTAGAATTCCAGGTGTAGCGGTGAAATGCGTAGAGATCTGGAGGAATACCGGTGGCGAAGGCGGCCCCCTGGACAAAGACTGACGCTCAGGTGCGAAAGCGTGGGGAGCAAACAGG
ASV3674	3	Lactobacillales	Levilactobacillus	TACGTAGGTGGCAAGCGTTGTCCGGATTTATTGGGCGTAAAGCGAGCGCAGGCGGTTATTTAAGTCTGATGTGAAAGCCTTCGGCTTAACCGAAGAAGTGCATCGGAAACTGGATGACTTGAGTGCAGAAGAGGACAGTGGAACTCCATGTGTAGCGGTGGAATGCGTAGATATATGGAAGAACACCAGTGGCGAAGGCGGCTGTCTAGTCTGTAACTGACGCTGAGGCTCGAAAGCATGGGTAGCAAACAGG
ASV3637	3	Lactobacillales	Brochothrix	TACGTAGGTGGCAAGCGTTGTCCGGAATTATTGGGCGTAAAGCGCGCGCAGGCGGTCTCTTAAGTCTGATGTGAAAGCCCCCGGCTCAACCGGGGAGGGTCATTGGAAACTGGGAGACTTGAGGACAGAAGAGGAGAGTGGAATTCCAAGTGTAGCGGTGAAATGCGTAGATATTTGGAGGAACA

				CCAGTGGCGAAGGCGGCTCTCTGGTCTGTTACTGACGCTGAGGCGCGAAAGCGTGGGGAGCAAACAGG
ASV3495	3	Burkholderiales	GKS98 freshwater group	TACGTAGGGTGCAAGCGTTAATCGGAATTACTGGGCGTAAAGCGTGCGCAGGCGGTTCGGAAAGAAAGATGTGAAATCCCAGAGCTCAACTTTGGAACTGCATTTTTAACTACCGGACTAGAGTGTGTCAGAGGGGGGTGGAATTCCGCGTGTAGCAGTGAAATGCGTAGAGATGCGGAGGAACACCGATGGCGAAGGCAGCCCCCTGGGATAACACTGACGCTCATGTACGAAAGCGTGGGTAGCAAACAGG
ASV1603	3	Flavobacteriales	Chishuiella	TACGGAGGGTGCAAGCGTTATCCGGATTTATTGGGTTTAAAGGGTCCGTAGGCGGATTAATCAGTCAGTGGTGAAATCCCTTAGCTTAACTAAGGAACTGCCATTGATACTGTTAGTCTTGAGTGAGGTTGCAGTGGCTGGAATGTGTAGTGTAGCGGTGAAATGCTTAGATATTACGCAGAACACCAATTGCGAAGGCAGGTCACTAAGCCTCAACTGACGCTGATGGACGAAAGCGTGGGGAGCGAACAGG
ASV1520	3	Xanthomonadales	Xanthomonas	TACGAAGGGTGCAAGCGTTACTCGGAATTACTGGGCGTAAAGCGTGCGTAGGTGGTGGTTTAAGTCTGTTGTGAAAGCCCTGGGCTCAACCTGGGAATTGCAGTGGATACTGGATCACTAGAGTGTGGTAGAGGGTAGCGGAATTCCCGGTGTAGCAGTGAAATGCGTAGAGATCGGGAGGAACATCCGTGGCGAAGGCGGCTACCTGGACCAACACTGACACTGAGGCACGAAAGCGTGGGGAGCAAACAGG
ASV91	3	Staphylococcales	*NA*	TACGTAGGTGGCAAGCGTTATCCGGAATTATTGGGCGTAAAGCGCGCGTAGGCGGTTTCTTAAGTCTGATGTGAAAGCCCACGGCTCAACCGTGGAGGGTCATTGGAAACTGGGAAACTTGAGTGCAGAAGAGGAGAGTGGAATTCCATGTGTAGCGGTGAAATGCGCAGAGATATGGAGGAACACCAGTGGCGAAGGCGGCTCTCTGGTCTGTAACTGACGCTGATGTGCGAAAGCGTGGGGATCAAACAGG
ASV297	3	Burkholderiales	Lampropedia	TACGTAGGGTGCAAGCGTTAATCGGAATTACTGGGCGTAAAGCGTGCGCAGGCGGCTATGCAAGACTGATGTGAAATCCCCGGGCTCAACCTGGGAACTGCATTAGTGACTGCATAGCTGGAGTGCGGCAGAGGGGGATGGAATTCCGCGTGTAGCAGTGAAATGCGTAGATATGCGGAGGAACACCGATGGCGAAGGCAATCCCCTGGGCCTGCACTGACGCTCATGCACGAAAGCGTGGGGAGCAAACAGG
ASV106	3	Staphylococcales	*NA*	TACGTAGGTGGCAAGCGTTATCCGGAATTATTGGGCGTAAAGCGCGCGTAGGCGGTTTCTTAAGTCTGATGTGAAAGTCCACGGCTCAACCGTGGAAGGTCATTGGAAACTGGGGAACTTGAGTGCAGAAGAGGAGAGTGGAATTCCATGTGTAGCGGTGAAATGCGCAGAGATATGGAGGAACACCAGTGGCGAAGGCGGCTCTCTGGTCTGTAACTGACGCTGATGTGCGAAAGCGTGGGGATCAAACAGG
ASV619	3	Enterobacterales	Providencia	TACGGAGGGTGCAAGCGTTAATCGGAATTACTGGGCGTAAAGCGCACGCAGGCGGTTGATTAAGTTAGATGTGAAATCCCCGGGCTTAACCTGGGAATGGCATCTAAGACTGGTCAGCTAGAGTCTTGTAGAGGGGGGTAGAATTCCATGTGTAGCGGTGAAATGCGTAGAGATGTGGAGGAATACCGGTGGCGAAGGCGGCCCCCTGGACAAAGACTGACGCTCAGGTGCGAAAGCGTGGGGAGCAAACAGG
ASV3714	2	Lactobacillales	Levilactobacillus	TACGTAGGTGGCAAGTGTTGTCCGGATTTATTGGGCGTAAAGCGAGCGCAGGCGGTTATTTAAGTCTGATGTGAAAGCCTTCGGCTTAACCGAAGAAGTGCATCGGAAACTGGATGACTTGAGTGCAGAAGAGGACAGTGGAACTCCATGTGTAGCGGTGGAATGCGTAGATATATGGAAGAACACCAGTGGCGAAGGCGGCTGTCTAGTCTGTAACTGACGCTGAGGCTCGAAAGCATGGGTAGCAAACAGG
ASV3625	2	Lactobacillales	Companilactobacillus	TACGTAGGTGGCAAGCGTTATCCGGATTTATTGGGCGTAAAGCGAGTGCAGGCGGTTTATTAGGTCTGATGTGAAAGCCCTCGGCTCAACCGAGGAAGTGCATCGGAAACCGGTAAACTTGAGTGCAGAAGAGGAGAGTGGAACTCCATGTGTAGCGGTGGAATGCGTAGATATATGGAAGAACACCAGTGGCGAAGGCGGCTCTCTGGTCTGTAACTGACGCTGAGGCTCGAAAGCGTGGGTAGCAAACAGG
ASV3090	2	Enterobacterales	*NA*	TACGGAGGGTGCAAGCGTTAATCGGAATGACTGGGCGTAAAGCGCACGCAGGCGGTTTGTTAAGTCAGATGTGAAATCCCCGAGCTTAACTTGGGAACTGCATTTGAAACTGGCAAGCTAGAGTCTTGTAGAGGGGGGTAGAATTCCAGGTGTAGCGGTGAAATGCGTAGAGATCTGGAGGAATACCGGTGGCGAAGGCGGCCCCCTGGACAAAGACTGACGCTCAGGTGCGAAAGCGTGGGGAGCAAACAGG
ASV1611	2	Acetobacterales	Gluconobacter	TACGAAGGGGGCTAGCGTTGCTCGGAATGACTGGGCGTAAAGGGCGCGTAGGCGGTTGATGCAGTCAGATGTGAAATCCCCGGGCTTAACCTGGGAACTGCATTTGAGACGCATTGACTAGAGTTCGAGAGAGGGTTGTGGAATTCCCAGTGTAGAGGTGAAATTCGTAGATATTGGGAAGAACACCGGTGGCGAAGGCGGCAACCTGGCTCGATACTGACGCTGAGGCGCGAAAGCGTGGGGAGCAAACAGG

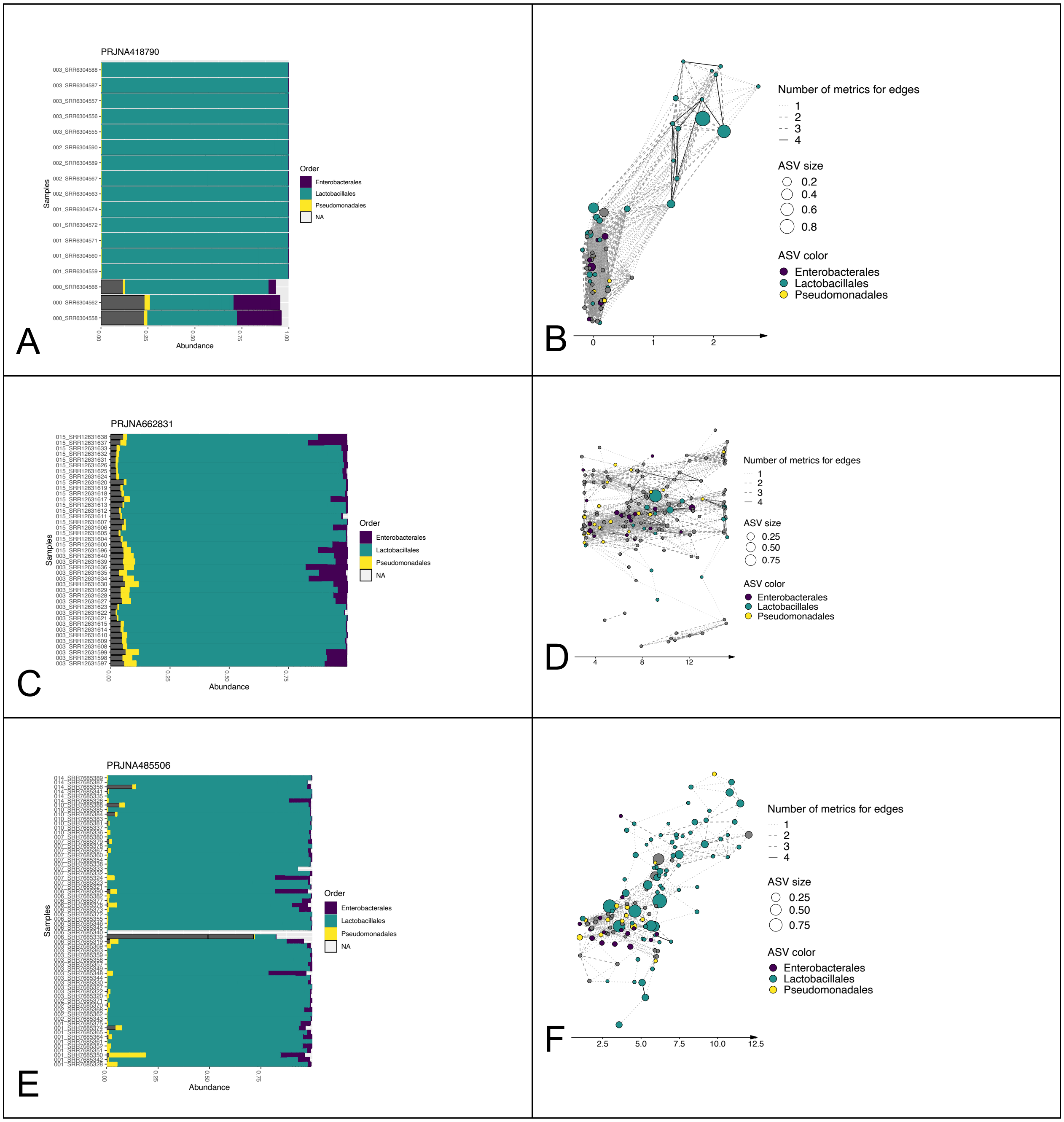

Figure S1: Microbial association networks highlight the dynamic evolution of microbial communities during fermentation. (A), (C), (E): Barplots depicting relative abundances in each sample for each study. Samples are ordered by age (the sampling time in days is included in the sample name). A dark gray color indicates a taxonomic order other than *Enterobacterales*, *Lactobacillales*, and *Pseudomonadales*, and NA corresponds to ASVs with unknown taxonomic affiliation at the order level. (B), (D), (F): ASV association networks for each study. Each node represents an ASV; node size reflects its maximum relative abundance and color represents its taxonomic order. The x-axis corresponds to the weighted mean age (WMA) of the samples in which the ASV was detected, measured in days, and weighted by ASV relative abundance. An edge between two nodes indicates an association that was detected according to at least one metric.

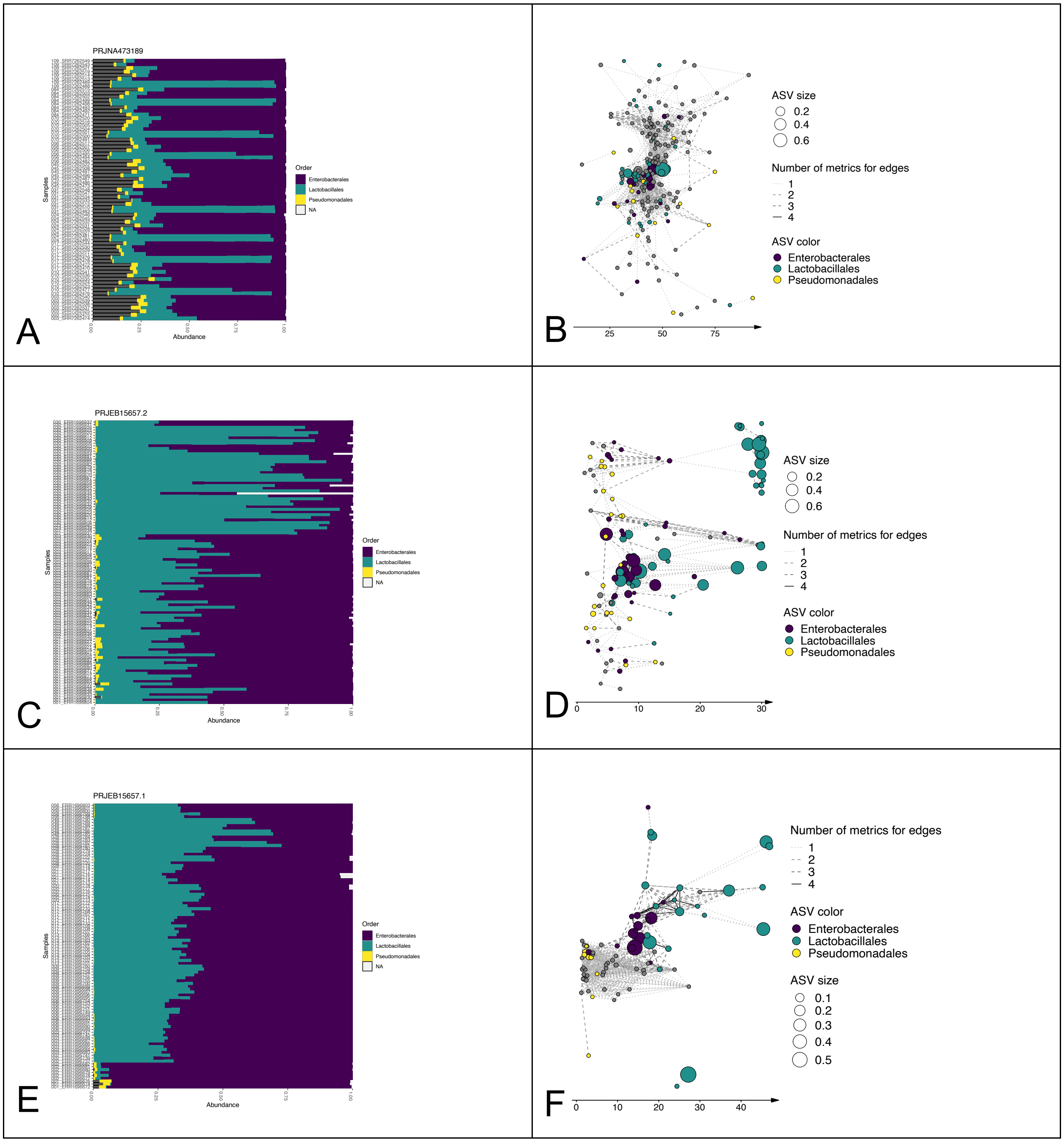

Figure S2: Microbial association networks highlight the dynamic evolution of microbial communities during fermentation. (A), (C), (E): Barplots depicting relative abundances in each sample for each study. Samples are ordered by age (the sampling time in days is included in the sample name). A dark gray color indicates a taxonomic order other than *Enterobacterales*, *Lactobacillales*, and *Pseudomonadales*, and NA corresponds to ASVs with unknown taxonomic affiliation at the order level. (B), (D), (F): ASV association networks for each study. Each node represents an ASV; node size reflects its maximum relative abundance and color represents its taxonomic order. The x-axis corresponds to the weighted mean age (WMA) of the samples in which the ASV was detected, measured in days, and weighted by ASV relative abundance. An edge between two nodes indicates an association that was detected according to at least one metric.

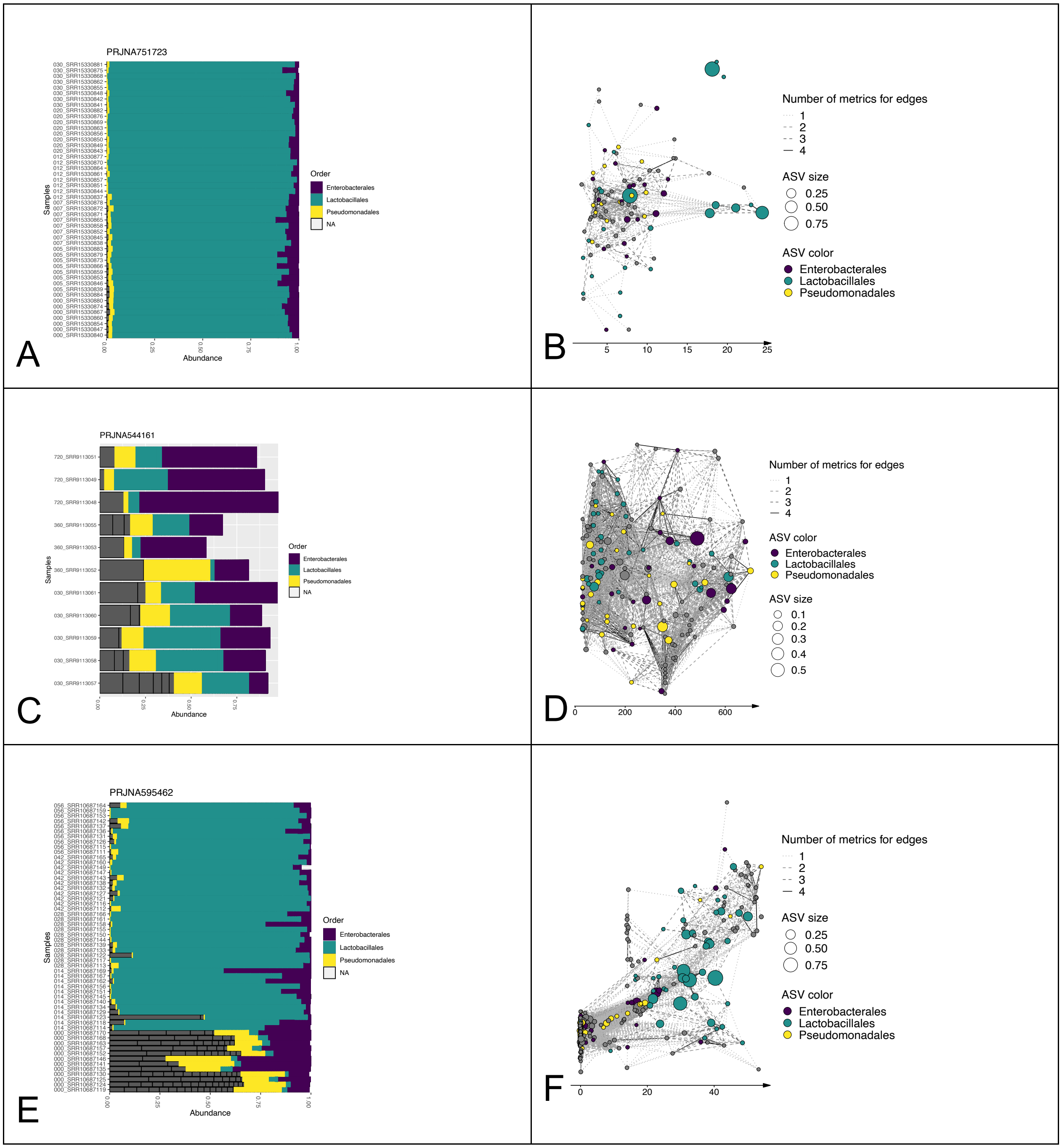

Figure S3: Microbial association networks highlight the dynamic evolution of microbial communities during fermentation. (A), (C), (E): Barplots depicting relative abundances in each sample for each study. Samples are ordered by age (the sampling time in days is included in the sample name). A dark gray color indicates a taxonomic order other than *Enterobacterales*, *Lactobacillales*, and *Pseudomonadales*, and NA corresponds to ASVs with unknown taxonomic affiliation at the order level. (B), (D), (F): ASV association networks for each study. Each node represents an ASV; node size reflects its maximum relative abundance and color represents its taxonomic order. The x-axis corresponds to the weighted mean age (WMA) of the samples in which the ASV was detected, measured in days, and weighted by ASV relative abundance. An edge between two nodes indicates an association that was detected according to at least one metric.

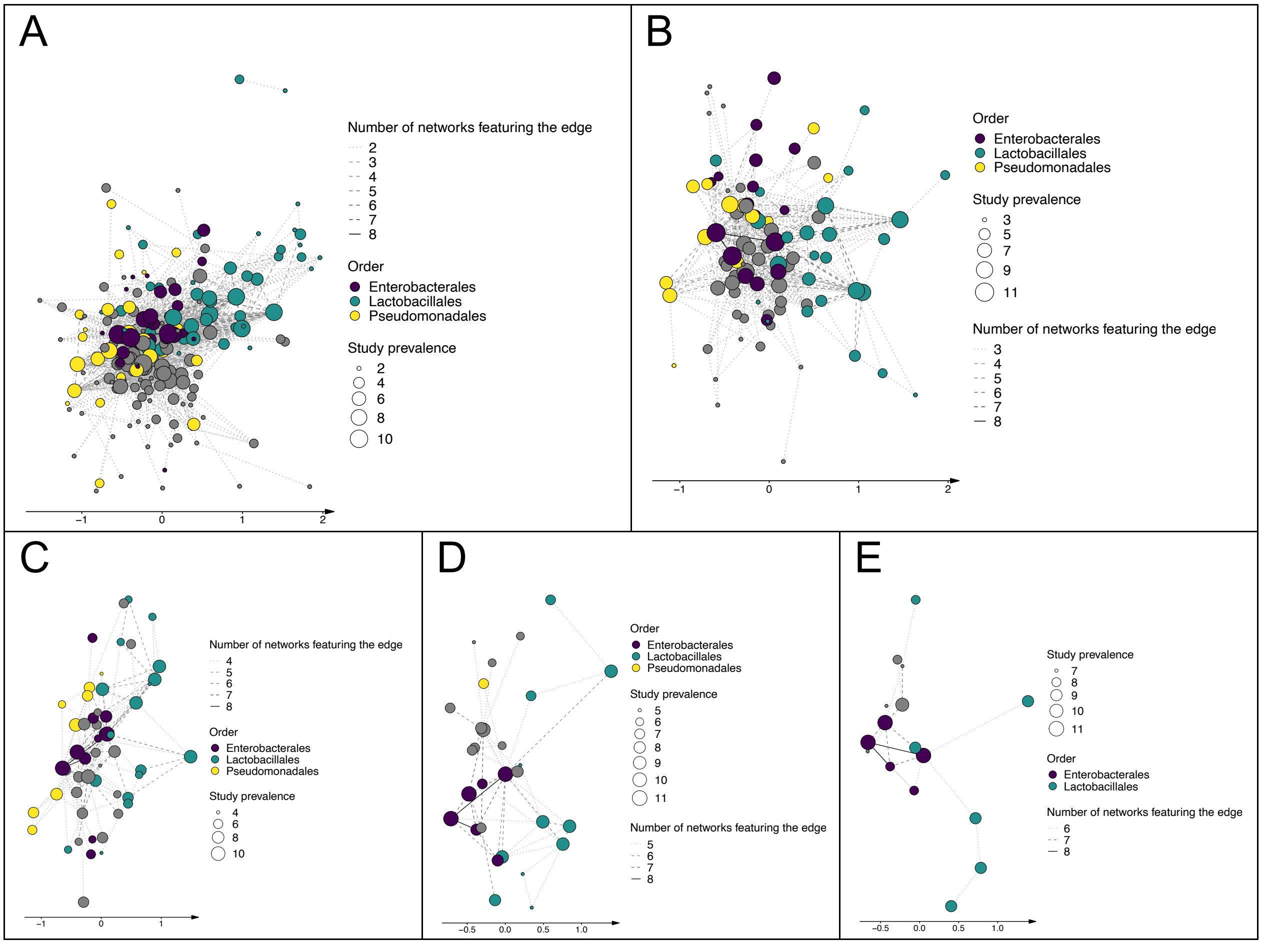

Figure S4: Core networks based on the intersections between two (A) to six networks (E). The line type of an edge represents the number of times the ASV association was found. The node position on the x-axis is the mean scaled WMA. ASVs are colored by taxonomic order.
